# Breakdown of the Static Dielectric Screening Approximation of Coulomb Interactions in Atomically Thin Semiconductors

**DOI:** 10.1021/acsnano.4c11563

**Published:** 2025-01-21

**Authors:** Amine Ben Mhenni, Dinh Van Tuan, Leonard Geilen, Marko M. Petrić, Melike Erdi, Kenji Watanabe, Takashi Taniguchi, Seth Ariel Tongay, Kai Müller, Nathan P. Wilson, Jonathan J. Finley, Hanan Dery, Matteo Barbone

**Affiliations:** † Walter Schottky Institute and TUM School of Natural Sciences, Technical University of Munich, 85748 Garching, Germany; ‡ Munich Center for Quantum Science and Technology (MCQST), 80799 Munich, Germany; § Department of Electrical and Computer Engineering, University of Rochester, Rochester, New York 14627, United States; ∥ Walter Schottky Institute and TUM School of Computation, Information and Technology, Technical University of Munich, 85748 Garching, Germany; ⊥ School for Engineering of Matter, Transport and Energy, Arizona State University, Tempe, Arizona 85287, United States; # Research Center for Electronic and Optical Materials, National Institute for Materials Science, 1-1 Namiki, Tsukuba 305-0044, Japan; ∇ Research Center for Materials Nanoarchitectonics, National Institute for Materials Science, 1-1 Namiki, Tsukuba 305-0044, Japan; ○ Department of Physics and Astronomy, University of Rochester, Rochester, New York 14627, United States

**Keywords:** transition metal dichalcogenides (TMDs), dielectric screening in 2D semiconductors, Coulomb interaction engineering, dynamical dielectric screening effects, high-dielectric-constant (high-K) materials, excitonic properties in van der Waals heterostructures, bandgap modulation

## Abstract

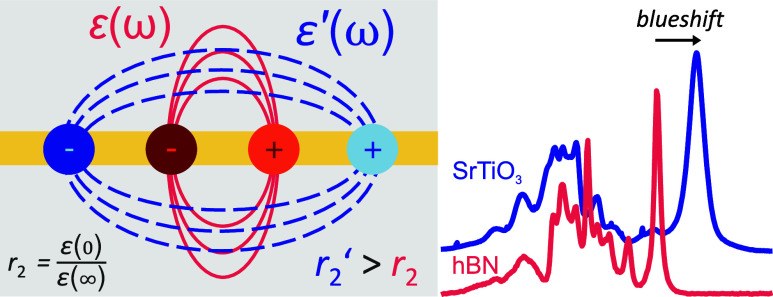

Coulomb interactions in atomically thin materials are remarkably sensitive to variations in the dielectric screening of the environment, which can be used to control exotic quantum many-body phases and engineer exciton potential landscapes. For decades, static or frequency-independent approximations of the dielectric response, where increased dielectric screening is predicted to cause an energy redshift of the exciton resonance, have been sufficient. These approximations were first applied to quantum wells and were more recently extended with initial success to layered transition metal dichalcogenides (TMDs). Here, we use charge-tunable exciton resonances to investigate screening effects in TMD monolayers embedded in materials with low-frequency dielectric constants ranging from 4 to more than 1000, a range of 2 orders of magnitude larger than in previous studies. In contrast to the redshift predicted by static models, we observe a blueshift of the exciton resonance exceeding 30 meV in higher dielectric constant environments. We explain our observations by introducing a dynamical screening model based on a solution to the Bethe-Salpeter equation (BSE). When dynamical effects are strong, we find that the exciton binding energy remains mostly controlled by the low-frequency dielectric response, while the exciton self-energy is dominated by the high-frequency one. Our results supplant the understanding of screening in layered materials and their heterostructures, introduce a knob to tune selected many-body effects, and reshape the framework for detecting and controlling correlated quantum many-body states and designing optoelectronic and quantum devices.

## Introduction

Interactions among particles give rise to collective phenomena described by new fundamental laws beyond simplified single-particle systems.^1^ This is particularly evident in heterostructures of two-dimensional (2D) materials, in which a wide variety of correlated electronic and excitonic phases have been realized, driven by strong Coulomb interactions.^2−6^ For instance, excitonic complexes up to eight particles^7−9^ and signatures of Wigner crystals^10^ have recently been reported in encapsulated, gated monolayer transition metal dichalcogenides (TMDs). Hubbard physics,^11,12^ unconventional superconductivity,^13^ and Chern insulators^14^ have been observed in moiré superlattices.

In all such phenomena, Coulomb interactions are heavily influenced by the dielectric response of the environment because the electric field generated by charged quasiparticles in a 2D material extends into the surrounding medium, which usually provides lower dielectric screening.^15−19^ This, in turn, leads to large exciton binding energy and single particle bandgap renormalization (BGR) effects. Therefore, Coulomb interaction engineering in atomically thin materials attracted considerable interest as a deterministic, scalable, and clean route to control many-body states, from exciton localization and transport to tuning many-body interactions in correlated states.^20−23^ To describe screening in semiconductor quantum wells and 2D materials, a common practice is to use an effective dielectric constant, neglecting frequency dependence and greatly simplifying the description of interactions between quasiparticles. Such description varies significantly depending on the screening weight attributed to the semiconductor and the environment layers.^24−29^ Theoretical investigations explored the impact of dynamical screening effects on the optical resonances in TMDs due to plasmons^30^ and optical phonons^31^ from the surrounding environment. However, within the limit of small variations to the dielectric constants (below an order of magnitude) of the environments and the carrier density studied so far, dynamical screening effects were predicted to introduce corrections to the binding energy and BGR,^30,31^ but did not appear to qualitatively alter the description of excitons in TMD monolayers.^18,20,32−36^

Here, we track gate-tunable exciton resonances in monolayer WSe_2_ embedded in environments with low-frequency dielectric constants ε(ω = 0) spanning 3 orders of magnitude but with high (optical)-frequency dielectric constants ε(ω = *∞*) changing by less than two times. In contrast with the preceding literature, we surprisingly observe an exciton resonance *blueshift* for larger dielectric constant environments, incompatible with the established theoretical understanding. We explain this behavior by introducing a model that accounts for the dynamic screening of electron–hole bound states, which shows that when dynamic effects are strong, the exciton binding energy primarily responds to ε(0), while the self-energy (the energy accounting for all interactions) of the bound state primarily depends on ε(*∞*). Crucially, the free-particle bandgap remains dependent on the low-frequency dielectric constant and manifests its inadequacy to describe bound electron–hole pairs under more extreme screening. Our results reveal conditions under which the frequency-independent dielectric screening approximation breaks down, and dynamical effects become a key factor in determining excitonic behavior. Furthermore, they indicate the necessity of including both dynamical screening effects and a bound-state description of excitons to fully capture screening effects in 2D systems. Materials with strong frequency-dependent dielectric functions allow the selective tuning of exciton binding energy and self-energy in atomically thin semiconductors, providing a knob to control quantum many-body states and their interactions and to design dielectrically engineered optoelectronic and quantum devices.

## Results and Discussion

### Effect of the Dielectric Screening on the Optical Spectrum of Monolayer TMDs

**Figure 1:**
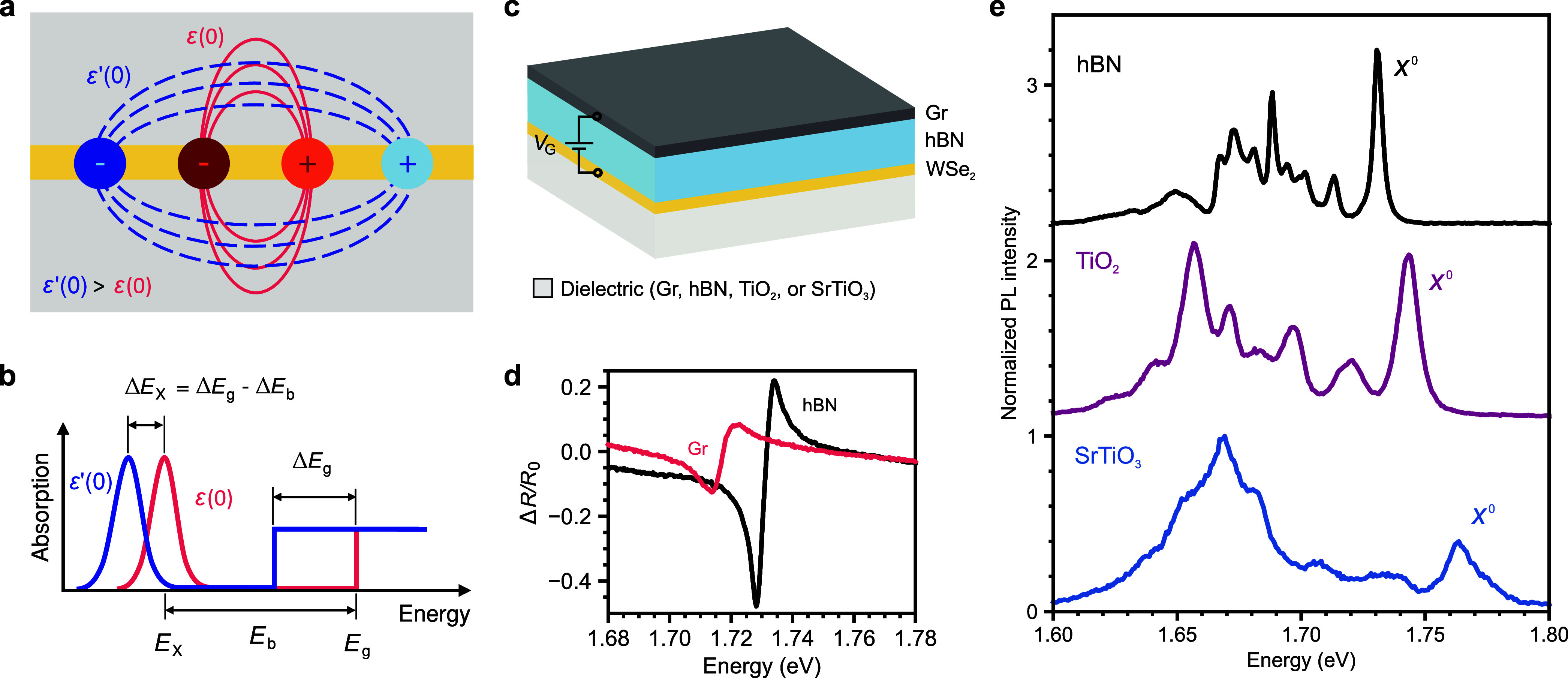
Dielectric screening in monolayer semiconductors and device configurations. (a) Schematic of an exciton and the electric field lines between its electron and hole when an atomically thin semiconductor is embedded in a weak (strong) screening environment ε(0) (ε*′*(0)). (b) Sketches of the absorption spectra expected from (a), with *E*_X_ being the energy resonance of the exciton ground state (*n* = 1), *E*_b_ the binding energy, and *E*_g_ the continuum single-particle bandgap energy (exciton energy in the limit *n* = *∞*). (c) Schematics of the gate-tunable devices employed in this study. In each device, a monolayer WSe_2_ is placed between hBN and a bottom dielectric, which is either Gr, hBN, TiO_2_, or SrTiO_3_. (d) Reflection contrast spectrum of an hBN (black) and of a Gr device (red) at 5 K. The resonance energy of *X*^0^ redshifts with increasing ε(0) of the environment. (e) Normalized PL spectra of the hBN, TiO_2_, and SrTiO_3_ devices at 8 K. Opposite to (d), the resonance energy of *X*^0^ blueshifts with increasing ε(0) of the environment.

Figure 1a shows the schematic of an exciton in an atomically thin semiconductor embedded in environments with two different effective ε(0) and ε′(0), where ε(0) < ε′(0). Exciton states manifest as discrete optical resonances below the renormalized free-particle bandgap energy, as shown in Figure 1b for the exciton ground state. The dielectric environment affects the exciton resonance energy through changes to both its binding energy and the electronic bandgap formed of the free electron and hole in the respective electronic bands, the latter being a BGR effect. For increasing effective ε(0), the bandgap reduces, inducing a *redshift* of the exciton resonance. At the same time, the binding energy also decreases, thereby inducing a *blueshift* of the exciton resonance. Scanning tunneling spectroscopy experiments, which measure the free-particle bandgap, and optical absorption, revealed the two effects to be of the same order of magnitude in TMDs (up to ∼ hundreds of meV), almost canceling each other.^19,20^ However, in the static approximation, the former is expected to be always slightly stronger than the latter by up to a few tens of meV.^29,37^ When calculating the BGR, the Coulomb potential Δ*V*(*r*) is evaluated at a distance *r* → 0, whereas the binding energy is evaluated at a finite distance. Since the difference between the Coulomb potentials in two dielectric environments is greatest at *r* = 0, the net effect should always be a redshift of the exciton resonance with increasing static dielectric constant.^29,37^ Importantly, this picture also implies that static screening alone does not allow independent tuning of binding energy and bandgap. To date, applications using dielectric engineering to control quasiparticles and their interactions as well as to design devices have rested on this understanding.

We fabricate charge-tunable devices based on monolayer WSe_2_ using van der Waals fabrication techniques (Methods). In this study, we use WSe_2_ as a prototypical TMD material since it offers a larger exciton Bohr radius than Mo-based TMDs,^35,38^ amplifying its sensitivity to the dielectric environment and because it does not display significant Fermi level pinning.^7,39^Figure 1c shows the device configuration. Monolayer WSe_2_ is sandwiched between a top layer of hexagonal boron nitride (hBN) and a bottom layer with varying ε(0), either hBN, few-layer graphene (Gr), TiO_2_, or SrTiO_3_. Throughout this work, we refer to the different dielectric screening configurations by their bottom layer. At temperatures ≤10 K, the effective ε(0) of these configurations goes from ∼3.5 for the hBN^10^ sample, to ∼7 for Gr,^40^ to ∼75 for the TiO_2_ sample,^41^ and >1000 for the SrTiO_3_ sample,^42^ spanning a range more than 2 orders of magnitude wider than previous studies.^18,20,32,35^ Gr is also used as a gate. Figure 1d compares the reflection contrast Δ*R*/*R*_0_ at charge neutrality for the Gr and hBN samples. Consistent with the conventional understanding previously discussed, the neutral exciton *X*^0^ energy redshifts about 15 meV from hBN to Gr due to the increasing ε(0).^20^Figure 1e shows the low-temperature photoluminescence (PL) spectra for hBN, TiO_2_, and SrTiO_3_ near charge neutrality, evidenced by the high ratio between the *X*^0^ and negative trion *X*^–^ intensities. In contrast to the Gr case as well as previous reports,^20,33,34^ the *X*^0^ energy surprisingly blueshifts with increasing effective ε(0), from 1.731 eV in the hBN device to 1.743 eV in the TiO_2_ device, and further to 1.764 eV in the SrTiO_3_ device. These findings are not limited to selected WSe_2_ samples, but we observe consistent blueshifts across more than 12 samples embedded in the same dielectric environments, also when replacing monolayer WSe_2_ with MoSe_2_ and WS_2_ (Supporting Information Figure S1).

**Figure 2:**
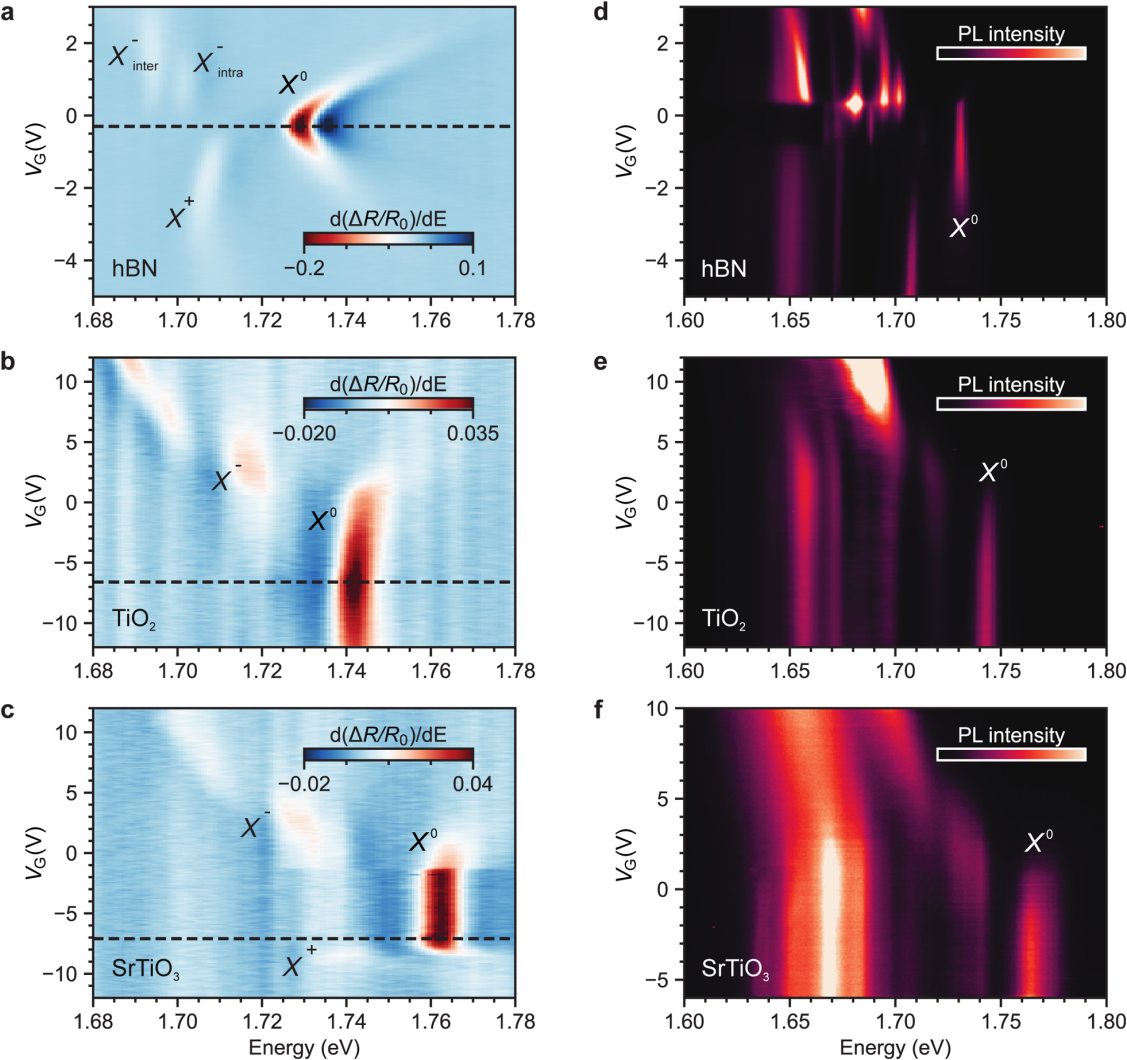
Gate-dependent optical response in ultrahigh ε(0) environments. (a–c) Gate-dependent d(Δ*R*/*R*_0_)/d*E* of monolayer WSe_2_ in the hBN (a), TiO_2_ (b), and SrTiO_3_ (c) dielectric configurations. The voltage corresponding to charge neutrality is indicated by a dashed horizontal line. (d–f) Gate-dependent PL spectra of monolayer WSe_2_ in the hBN (d), TiO_2_ (e), and SrTiO_3_ (f) dielectric configurations. In both measurements, at charge neutrality *X*^0^ blueshifts with increasing effective ε(0) of the environment.

In contrast with past studies, we measure the gate-dependent optical response of monolayer WSe_2_ in the different dielectric configurations to exclude possible contributions to the exciton resonance shift from charge doping.^43^Figure 2a–c compares the reflection contrast derivative d(Δ*R*/*R*_0_)/d*E* from the hBN, TiO_2_, and SrTiO_3_ samples. In all cases, we extract the *X*^0^ energy by fitting a dispersive Lorentzian at the charge neutrality point identified from the *X*^0^ absorption maximum (Supporting Information Figure S2). In the hBN sample (Figure 2a), the energy of *X*^0^ is 1.731 eV. The spectrum of *X*^0^ exhibits a pronounced broadening and an energy blueshift greater than 15 meV from charge neutrality to higher charge doping. This highlights the importance of evaluating excitonic energies at charge neutrality in such studies. The negative exchange-split trions^7^ (*X*_intra_^–^ and *X*_inter_^–^) appear in the electron-doped regime (positive *V*_G_). In contrast, the positively charged trion^7^ (*X*^+^) becomes visible in the hole-doped regime (negative *V*_G_). The TiO_2_ sample (Figure 2b) shows the *X*^0^ at 1.740 eV, 9 meV blueshifted with respect to *X*^0^ in the hBN sample. Even more, the SrTiO_3_ sample (Figure 2c) shows an *X*^0^ energy of 1.762 eV, 31 meV blueshifted with respect to that in the hBN sample.

To further corroborate our findings, we inspect the optical response of WSe_2_ via gate-dependent PL spectroscopy and extract the energy of *X*^0^ at charge neutrality. Figure 2d–f shows the gate-dependent PL spectra of the hBN, TiO_2_, and SrTiO_3_ samples. In the hBN sample (Figure 2d), the energy of *X*^0^ is 1.731 eV, accompanied by a line width below 2 meV, consistent with the highest quality samples reported in the literature,^7,39,44,45^ and blueshifts due to charge doping by up to 5 meV before disappearing (Supporting Information Figure S3). The excited states 2*s*, 3*s*, 4*s*, and (2*s*)^+^ are well-resolved in the PL spectra (Supporting Information Figure S4), further testifying to the high sample quality.^44,45^ In the TiO_2_ sample (Figure 2e), *X*^0^ has a line width of less than 5 meV and appears at 1.743 eV, blueshifted by ∼12 meV compared to that in the hBN sample. In the SrTiO_3_ sample (Figure 2f), *X*^0^ has a line width of ∼6 meV and arises at 1.764 eV, blueshifted by ∼33 meV compared to that in the hBN sample. The line width broadening may be a consequence of the coupling of excitons with the lower energy optical phonons of the substrates.^31^ Overall, the PL measurements are in good agreement with the reflection contrast data.

To exclude any contribution to the exciton energy shifts from uncontrolled strain fields^46^ or other spatially dependent effects, we study the *X*^0^ energy distribution over large areas on multiple samples for each dielectric configuration (Supporting Information Figure S5). We observe a narrow distribution below 3 meV, reflecting the high homogeneity of the samples and the repeatability of the observations.

Since ε^SrTiO_3_^ (0) increases over 1 order of magnitude between 100 and 5 K,^42^ we also look at the temperature dependence of the exciton resonance in the SrTiO_3_ device (Supporting Information Figure S7). Going from 80 K down to 20 K, *X*^0^ exhibits a blueshift (∼23 meV), which is more than twice as large as the blueshift in the hBN sample (∼9 meV). This observation is consistent with *X*^0^ blueshifting due to increasing ε(0) of the environment. Moreover, it unveils a new pathway to control *X*^0^ on the same device by tuning the ε(0) of SrTiO_3_ via temperature or via electric fields.^42^

### Fully Dynamical Description of Coulomb Screening

**Figure 3:**
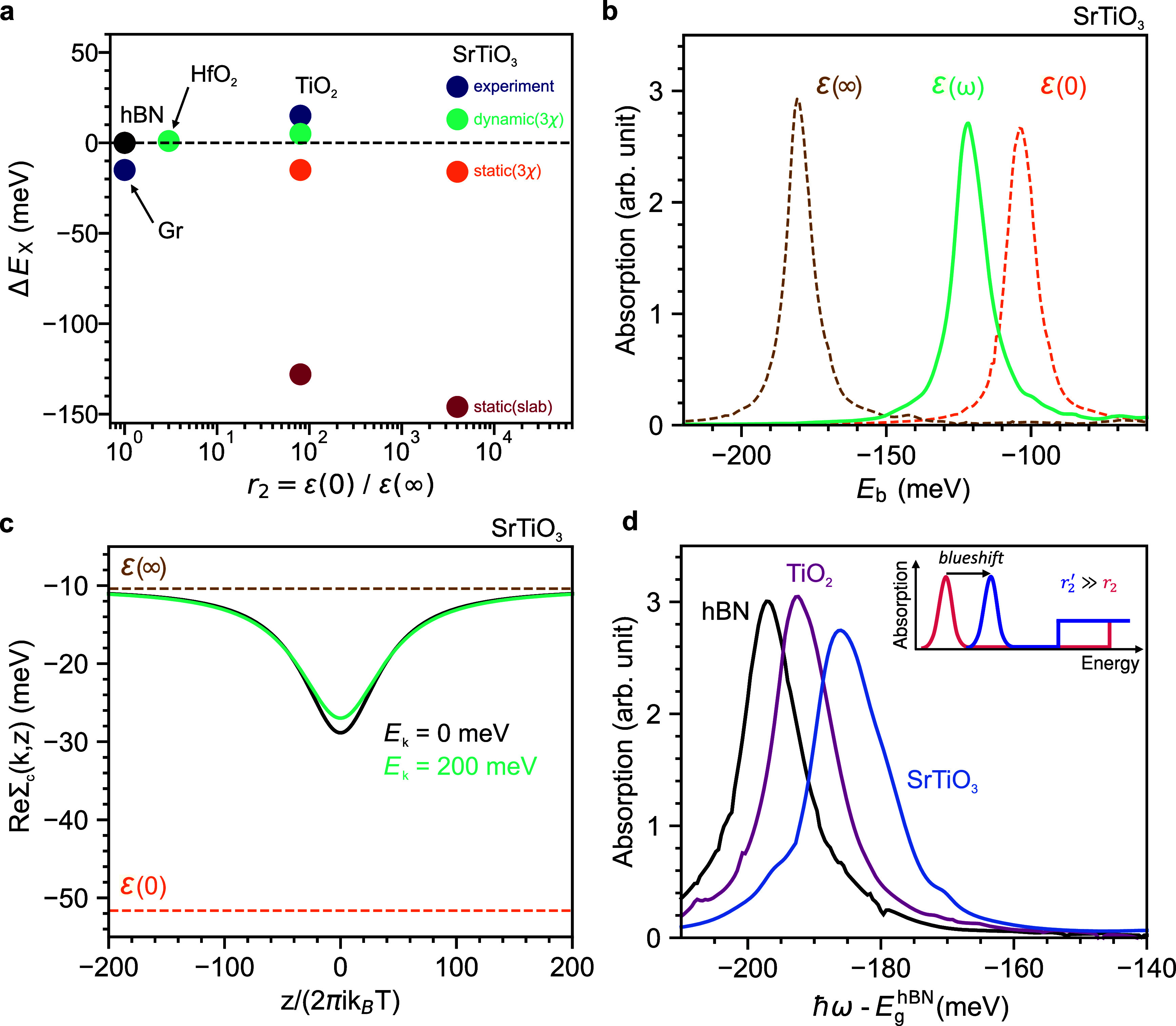
Modeling dynamical dielectric screening effects. (a) Comparison of the energy shift of *X*^0^ in monolayer WSe_2_ as a function of *r*_2_ from the optical experiments and from the theoretical calculations for both static and dynamical models. Static models always lead to redshift, with the slab model (red) diverging by almost 180 meV from the experimental results (blue), by almost 170 meV from the dynamical 3χ (green), and by almost 130 meV from the static 3χ (orange). (b) Exciton binding energy calculated from the absorption spectrum of monolayer WSe_2_ on SrTiO_3_ by neglecting the self-energy terms in the BSE equation and using ε(0) (orange), ε(*∞*) (brown), and ε(ω) (green). (c) Calculated real part of the single-particle self-energy Σ_c_(*k*, *z*) of conduction band electrons as a function of Matsubara frequencies for the SrTiO_3_ sample. Solid lines indicate dynamical calculations for two relevant electron energies *E*_*k*_, while dashed lines are the results of the single-particle BGR calculated with ε(*∞*) (brown) and ε(0) (orange). Zero energy is set to the calculated static self-energy of the hBN sample for ε(*∞*). (d) Absorption spectra corresponding to the samples measured experimentally calculated with dynamical screening (ε(ω)) by including both the dynamical potential and self-energies of the electron and hole in the exciton. The spectra are plotted relative to  eV. In the inset, schematic of the absorption spectrum of a monolayer TMD highlighting the exciton resonance blueshift for *r*_2_ increasing from ∼1 to ≫1.

We compare our experimental results with the theoretical predictions from two models employed to describe the influence of the environment on exciton resonances in TMDs in the static screening approximation, the “3χ” model^28^ and the “slab” model,^29^ and track the predicted exciton resonance shift with varying screening *r*_2_ = ε(0)/ε(*∞*) from the reference point of a top and bottom hBN environment. Figure 3a shows that with increasing *r*_2_, exciton resonances according to the 3χ and the slab model are expected to redshift from the hBN reference up to about 16 and 145 meV respectively, or about 45–170 meV lower in energy than our experimental results. The large difference between the two models stems from the lower screening weight attributed to the surrounding environment by the 3χ. For a homogeneous strain field to be the source of such a shift, that would amount to a compressive strain ∼1 to 4%,^47^ which has never been reported even for externally applied deformation, while the adhesion energy of WSe_2_ to the substrate would only support a planar strain well below 0.1% before delamination.^48^ Also, the energy of the ground state (1*s*) exciton resonance is less sensitive to strain than other established experimental routes, such as the relative energy between the 1*s* and the 2*s* exciton,^46^ which is employed as a more direct measurement of the binding energy and the electronic bandgap.^20^ Having excluded other potential sources of blueshift, we conclude that *X*^0^ blueshifts with an increasing static ε(0) of the environment. This implies that the corresponding reduction in the exciton binding energy must be greater than the BGR. Hence, the static approximation of Coulomb interactions is not sufficient to describe the dielectric screening in atomically thin semiconductors.

To reconcile the contradiction between our results and the theory of screened many-body interactions, we turn to examining the role of frequency dependence in dielectric screening. The response of a dielectric material to an electric field comes from its valence electrons and, if the material is polar, from field-induced lattice vibrations that induce a net atomic polarization.^49^ The electron and hole are not independent entities; instead, they move with respect to each other with kinetic energy commensurate with the exciton binding energy as dictated by the virial theorem, resulting in a varying electric field.^28,37^ Consequently, we lift the assumption that the atoms of the encapsulating layers either perfectly trace (ε(0)) or completely ignore (ε(*∞*)) the variation of the electric field. If the dielectric layer adjacent to the monolayer semiconductor is a polar material, we can approximate its response to the electric field at frequency ω by the dielectric function:
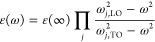
1

The ratio between the low- and high-frequency dielectric constants is the Lyddane-Sachs-Teller relation .^49^ The index *j* runs over the optical phonon modes, where ω_*j*,LO/TO_ is the associated frequency of the longitudinal/transverse optical lattice vibration in the dielectric layers. In the following, we use the 3χ formulation of the Coulomb potential^28^ and introduce dynamical dielectric functions to model the response of top and bottom dielectrics. We calculate the dynamical self-energies of the electron and hole in the exciton from the solution of the Dyson equation and use them for the BSE which we solve by an iterative method to obtain the absorption spectrum^50^ (Supporting Information).

Figure 3a shows the summary of the results for energy shift Δ*E*_*x*_ of the exciton resonance. Consistent with our measurements, *X*^0^ blueshifts because the binding energy blueshift contribution Δ*E*_b_ is larger than the redshift contribution Δ*E*_g_ for a higher *r*_2_. To understand the physical reasons behind such results, we consider individually each contribution.

Figure 3b shows the binding energy of the SrTiO_3_ sample calculated by neglecting the self-energy terms from the exciton Green function. The large difference between ε(0) and ε(*∞*) leads to a significant difference between the binding energy calculated employing ε(0), which assumes that atoms can readily trace the varying electric field of the electron and hole, and that calculated with ε(*∞*), which only considers the electronic contribution to the screening, with the former ∼80 meV smaller than the latter. Calculating the binding energy by using the dynamical dielectric function ε(ω) in the effective BSE,^51^ we obtain results closer to ε(0), indicating that in materials with large *r*_2_ like SrTiO_3_ the binding energy is mostly influenced by the low-frequency static dielectric response, while the opposite is true when *r*_2_ is close to 1.^52^Figure S5 shows the 2*s* energy peak measured from the PL spectrum of the SrTiO_3_ sample. The energy separation between the 1*s* and 2*s* excitons is lower than the corresponding value in the hBN sample, which implies a lower binding energy, consistent with the calculations.

Figure 3c shows the real part of the dynamic single-particle self-energy Σ_c_(*k*, *z*) of conduction band electrons in the SrTiO_3_ sample as a function of imaginary Matsubara frequencies. The reference point at 0 meV is set to the static self-energy calculated for the hBN sample with ε(*∞*). The self-energies calculated for the SrTiO_3_ sample at ε(0) and ε(*∞*) are ∼40 meV apart. We calculate the dynamical self-energy for two relevant electron energies *E* = *ℏ*^2^*k*^2^/2*m*_c_ of 0 and 200 meV. Across the whole Matsubara frequency spectrum, and in particular, for high frequencies, the self-energies remain close to the value calculated with ε(*∞*), indicating that the dynamical self-energy is mainly influenced by the high-frequency dielectric response. The calculation is performed with the bandgap *E*_g_ = 1.9 eV. Additional calculations with different bandgaps show that decreasing *E*_g_ makes the dynamical self-energy come closer to the low-frequency BGR, indicating that *E*_g_ plays an important role in the self-energies of the exciton components.^52^

Figure 3d presents the calculated absorption spectra of *X*^0^ by including both self-energy and dynamical potential in the BSE for all of the dielectric configurations considered in our experiments. The results show a net blueshift with increasing ε(0), in agreement with the experimental findings. To bridge the parameter gap between hBN and TiO_2_ and provide further guidelines to dielectric engineering efforts, we also calculate the optical resonance for an intermediate screening material system, hBN/WSe_2_/HfO_2_, having *r*_2_ ∼ 3,^53,54^ which we include in Figure 3a. The blueshift from the hBN reference energy is present, but it is found to be small, ∼1 meV. We provide all material parameters employed in the calculations in Supporting Information Table S1.

Despite the qualitative agreement of our theoretical and experimental results, we notice a lower shift in the calculations, possibly due to underestimation of the environmental screening in the 3χ model,^28^ as well as a possible smaller difference of the ε(*∞*) values (i.e. ε^SrTiO_3_^(*∞*) – ε^hBN^(*∞*) < 3.2). We also underline that we are unable to obtain a blueshift from the slab model even including dynamical screening: the much larger weight attributed to the environmental screening beyond the TMD layer always results in a dominant BGR term.

Our understanding also consistently bridges graphene with stronger dielectric screening materials. Unlike the case of TiO_2_ and SrTiO_3_, where strongly polar oxides result in extremely high ε(0), graphene is a nonpolar material, which is equivalent to having *r*_2_ → 1 (or ε(0) ≈ ε(*∞*)), with the large carrier mobility in graphene resulting in a very effective electronic screening. Our dynamic formalism based on the Lyddane-Sachs-Teller relation cannot be extended to metallic environments such as graphene, however, at the charge doping densities investigated in our work, dynamical effects due to plasmons are not expected to visibly affect the excitonic optical resonances.^30^ When dynamical effects become negligible, or equivalently *r*_2_ → 1, *r*_2_ ceases to be the primary parameter affecting exciton screening, supplanted by ε(0). Thus, we expect TMD excitons screened by Gr to redshift from the hBN reference energy, which we observed in Figure 1d. The value of the optical resonance of the Gr sample is included in Figure 3a. We stress that our findings stem from the effect of dynamical dielectric screening on the bound exciton: the self-energy of a bound exciton is not the self-energy of a free electron in the conduction band, plus that of a free hole in the valence band. In a bound pair, the bandgap energy introduces a relative phase exp(*iE*_g_*t*/*ℏ*) between the electron and hole components, and therefore, at least one of these components is influenced by the high-frequency dielectric response (Supporting Information Theoretical Methods). This subtle but key detail is lost if one considers only the self-energy of a free particle, which is influenced by ε(0) because the reference energy level, in this case, is the edge of the relevant energy band. This has important experimental consequences: if ε(0) is very different from ε(*∞*), single-particle electronic bandgap measurements such as ARPES or scanning tunneling spectroscopy^19^ provide incorrect results to derive the self-energy of a bound electron–hole pair. Our results also indicate that the exciton self-energy and the exciton binding energy can be individually controlled by selecting screening materials with different *r*_2_ values. Achieving the highest exciton energy difference at a dielectric heterojunction requires maximizing the Δ*r*_2_ values among the different dielectric materials and employing a low *r*_2_ material with highest ε(0).

### Effect of the Dielectric Screening on Short-Range Coulomb Interactions

**Figure 4:**
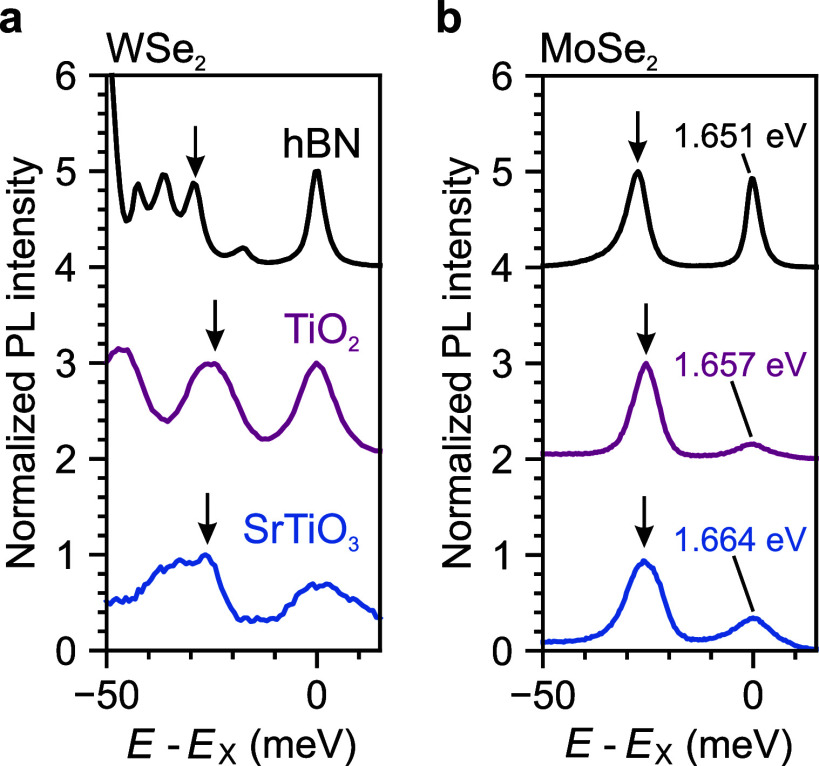
Effect of the dielectric environment on the trion binding energy. (a) PL spectra of monolayer WSe_2_ on hBN, TiO_2_, and SrTiO_3_ in the electron doping regime. *E*_X_ is taken as the origin of the energy axis. In WSe_2_, the negative trions are exchange-split. In each spectrum, *X*_intra_^–^ is indicated by a black arrow. (b) PL spectra of monolayer MoSe_2_ on hBN, TiO_2_, and SrTiO_3_ in the electron doping regime. E_X_ is taken as the origin of the energy axis and is indicated on the plots. *X*^–^ is indicated by a black arrow in each spectrum.

To understand the dielectric screening effects on many-body complexes beyond excitons, we also experimentally investigate the behavior of the trion. Figure 4a shows the PL spectra of monolayer WSe_2_ for the hBN, TiO_2_, and SrTiO_3_ samples in the electron doping regime, but close to charging neutrality (the *X*^0^ and negative trions intensities are comparable) to minimize energy shifts from charge doping. The exciton resonance *X*^0^ of each sample is taken as the origin of the energy axis to allow for a direct comparison of the trion binding energies across the different dielectric configurations. The negatively charged intravalley trion *X*_intra_^–^ shows only a weak dependence on *r*_2_. Its binding energy starts at ∼30 meV in the hBN sample, drops to ∼24 meV in the TiO_2_ sample, and rises to ∼27 meV in the SrTiO_3_ sample. The nonmonotonic behavior may be attributed to residual energy shifts from inconsistencies in charge doping among samples. To investigate the same effect in a material with spectrally well-separated resonances, we also study the *X*^–^ binding energy in monolayer MoSe_2_. Figure 4b shows the PL spectra of monolayer MoSe_2_ for the hBN, TiO_2_, and SrTiO_3_ samples in the electron doping regime. We first note that *X*^0^ in MoSe_2_ also experiences a blueshift of up to 13 meV at higher values of *r*_2_. However, we do not observe any meaningful *X*^–^ binding energy dependence on *r*_2_, with the change being of the order of only a few meV.

The weak sensitivity of the trion binding energy in WSe_2_ and MoSe_2_ to *r*_2_, together with the conservation of many of the excitonic features at extreme *r*_2_ values, suggests that the formation of trions and other excitonic few-body complexes is only weakly affected by static ε(0). At large distances, the interaction between a neutral exciton and an extra charge is dipolar in nature and, thus, has a relatively fast decay (*V*(*r*) ∼ 1/*r*^2^). Consequently, the binding energy of few-body complexes such as the trion is governed by short-range interparticle interactions, which are not sensitive to low-frequency screening.^28^

## Conclusions

Coulomb interactions in atomically thin semiconductors coupled to polar oxides require a physical description beyond the static dielectric constant approximation, breaking the monolithic picture of exciton binding energy and BGR as effects governed by the same type of screening and revealing a nuanced interplay of phenomena with a distinct frequency dependence. Our results offer new avenues to study and manipulate many-body interactions and provide the necessary physical understanding to predict exciton behavior when integrating TMDs and functional oxides. A natural consequence of our work would be to couple states with built-in electrical dipoles with polar oxides, such as Janus TMDs,^55,56^ or to tune interlayer and moiré excitons via the dielectric environment. Using excitonic resonances as sensors for charge ordering could provide deeper insights into correlated states. An exciting direction would be to explore the tuning of long-range interactions in strongly correlated systems, for example, in systems realizing the extended Hubbard model. This may allow the realization of currently inaccessible many-body phases, including interaction-induced Chern insulators and quantum spin liquids.^57,58^ Finally, enabling the deterministic fabrication of dielectric superlattices could unlock the study of strongly correlated physics in artificial solid-state crystals and quasicrystals.^21^

## Methods and Experimental Section

### Sample Preparation

All of the TMD, Gr, and hBN flakes were mechanically exfoliated from bulk crystals on SiO_2_ substrates. The flakes were selected based on their optical contrast, shape, and cleanliness. Single-crystal substrates of (001) TiO_2_ (Rutile phase) and (100) SrTiO_3_ were acquired from Shinkosha Co., Ltd. Oxide single-crystals have a purity >99.98% and RT ε(0) measured at 1 MHz of 113 and 300, respectively. The devices were assembled via dry-transfer technique using polycarbonate films^59^ for the hBN and TiO_2_ devices and polypropylene carbonate^60^ for the SrTiO_3_ devices. Contacts to the respective layers were patterned using optical lithography and electron beam evaporation (Cr/Au 5/100 nm).

### Optical Spectroscopy

Optical measurements were performed in a variable-temperature helium flow cryostat with a confocal microscope in a reflection geometry. For the PL measurements, 633 nm/532 nm continuous wave laser sources were used for the excitation. The laser beam was focused onto the sample using an objective with a numerical aperture of 0.75, yielding an excitation spot size of around 1 μm. A pinhole was used as a spatial filter to obtain a diffraction-limited collection spot. The collected light is dispersed using a grating monochromator and detected on a CCD sensor array. The laser light was filtered by using a 650/550 nm short-pass filter. For reflection contrast spectroscopy, thermal light from a tungsten halogen light source was used for excitation. The gate voltage in the gate-tunable measurements was controlled by using a Keithley 2400 source meter. Unless otherwise specified, all measurements presented here were performed at 10 K. A close-cycle optical cryostat in reflection geometry (Attocube, attoDRY800) with variable-temperature capability was used to perform the temperature-dependent measurements presented in the Supporting Information.

## Data Availability

The data sets generated and analyzed during the current study are available from the corresponding authors upon reasonable request.

## Supporting Information Available

Effect of the dielectric screening on *X*^0^ in MoSe_2_ and WS_2_; insights in the reflection contrast and PL data analysis; Effect of charge doping on *E*_X_ for monolayer WSe_2_ in the hBN sample; PL of the exciton Rydberg series of monolayer WSe_2_ in the hBN sample; Differential reflectance of the exciton Rydberg series of monolayer WSe_2_ in the SrTiO_3_ sample; *X*^0^ exciton energy distribution from PL sample maps; temperature-dependence of the SrTiO_3_ sample; and theoretical methods (PDF)

## Notes

An early version of this manuscript was submitted as preprint: Ben Mhenni, A.; Van Tuan, D.; Geilen, L.; Petrić, M. M.; Erdi, M.; Watanabe, K.; Taniguchi, T.; Tongay, S.; Müller, K.; Wilson, N. P.; Finley, J. J.; Dery, H.; Barbone, M. Breakdown of the Static Dielectric Screening Approximation of Coulomb Interactions in Atomically Thin Semiconductors. 2024, arXiv:2402.18639. ArXiv e-prints. https://doi.org/10.48550/arXiv.2402.18639 (28 Feb 2024).
