# Supplementary material for: Breakdown of the Static Dielectric Screening Approximation of Coulomb Interactions in Atomically Thin Semiconductors
Source: ACS Nano. 2025 Jan 21;19(4):4269–78. doi: 10.1021/acsnano.4c11563 (PMC11803920; doi:10.1021/acsnano.4c11563)
Supplement: Supplementary file 1 — nn4c11563_si_001.pdf [file nn4c11563_si_001.pdf]

Supporting Information for: Breakdown of the static dielectric screening approximation of Coulomb interactions in atomically thin semiconductors

Amine Ben Mhenni,[*,†,‡] Dinh Van Tuan,[¶] Leonard Geilen,[†,‡] Marko M. Petrić,[§,‡] Melike Erdi,[‖] Kenji Watanabe,[⊥] Takashi Taniguchi,[#] Seth Ariel Tongay,[‖] Kai Müller,[§,‡] Nathan P. Wilson,[†,‡] Jonathan J. Finley,[*,†,‡] Hanan Dery,[¶,@] and Matteo Barbone[*,§,‡]

†*Walter Schottky Institute and TUM School of Natural Sciences, Technical University of Munich, 85748 Garching, Germany*

‡*Munich Center for Quantum Science and Technology (MCQST), 80799 Munich, Germany*

¶*Department of Electrical and Computer Engineering, University of Rochester, Rochester, New York 14627, USA*

§*Walter Schottky Institute and TUM School of Computation, Information and Technology, Technical University of Munich, 85748 Garching, Germany*

‖*School for Engineering of Matter, Transport and Energy, Arizona State University, Tempe, Arizona 85287, USA*

⊥*Research Center for Electronic and Optical Materials, National Institute for Materials Science, 1-1 Namiki, Tsukuba 305-0044, Japan*

#*Research Center for Materials Nanoarchitectonics, National Institute for Materials Science, 1-1 Namiki, Tsukuba 305-0044, Japan*

@*Department of Physics and Astronomy, University of Rochester, Rochester, New York 14627, USA*

E-mail: amine.ben-mhenni@tum.de; jj.finley@tum.de; matteo.barbone@wsi.tum.de

Contents

S1 Blueshift of X^0 in $MoSe_2$ and WS_2 **3**

S2 Optical Data Analysis **4**

S3 Blueshift of X^0 Due to Charge Doping **5**

S4 Rydberg Series of WSe_2 in the hBN Device **6**

S5 $2s$ Exciton in an $SrTiO_3$ Device **7**

S6 Neutral Exciton Energy Distribution **9**

S7 Temperature-dependence of the $SrTiO_3$ Device and Saturation of the Dielectric Effect **10**

S8 Theoretical methods **12**

S8.1 Theory 12

S8.2 Dynamical Self-Energy 13

S8.3 Dynamical Bethe-Salpeter Equation 15

S8.4 Distinction between static and dynamical calculations 16

S8.5 Dynamical effects in the optical spectrum 17

S8.6 Iterative method 18

S8.7 Material parameters 18

References **20**

S1 Blueshift of X^0 in $MoSe_2$ and WS_2

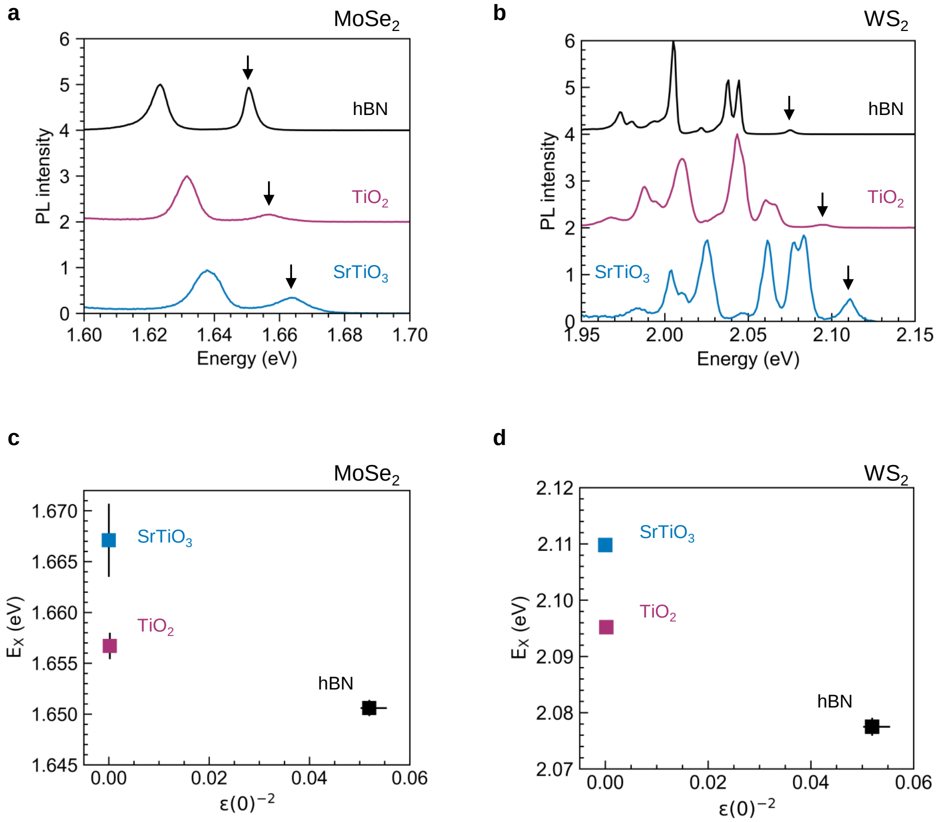

Figure S1. Effect of the dielectric screening on X^0 in $MoSe_2$ and WS_2. (a) PL spectra of ungated monolayer $MoSe_2$ in the hBN, TiO_2, and $SrTiO_3$ dielectric configurations. (b) PL spectra of ungated monolayer WS_2 in the hBN, TiO_2, and $SrTiO_3$ dielectric configurations. The spectral position of X^0 is indicated by an arrow in both figures. Both for $MoSe_2$ and WS_2, X^0 blueshifts with a higher static dielectric constant. Here, the shift resulting from charge doping was not accounted for. However, the magnitude of the blueshift is much larger than what can be explained by just charge doping effects. (c) and (d) E_X as a function of $\varepsilon(0)^{-2}$ for $MoSe_2$ (c) and WS_2 (d). The mean value of E_X across large areas on more than one sample was taken for each data point. The standard deviation is plotted.

S2 Optical Data Analysis

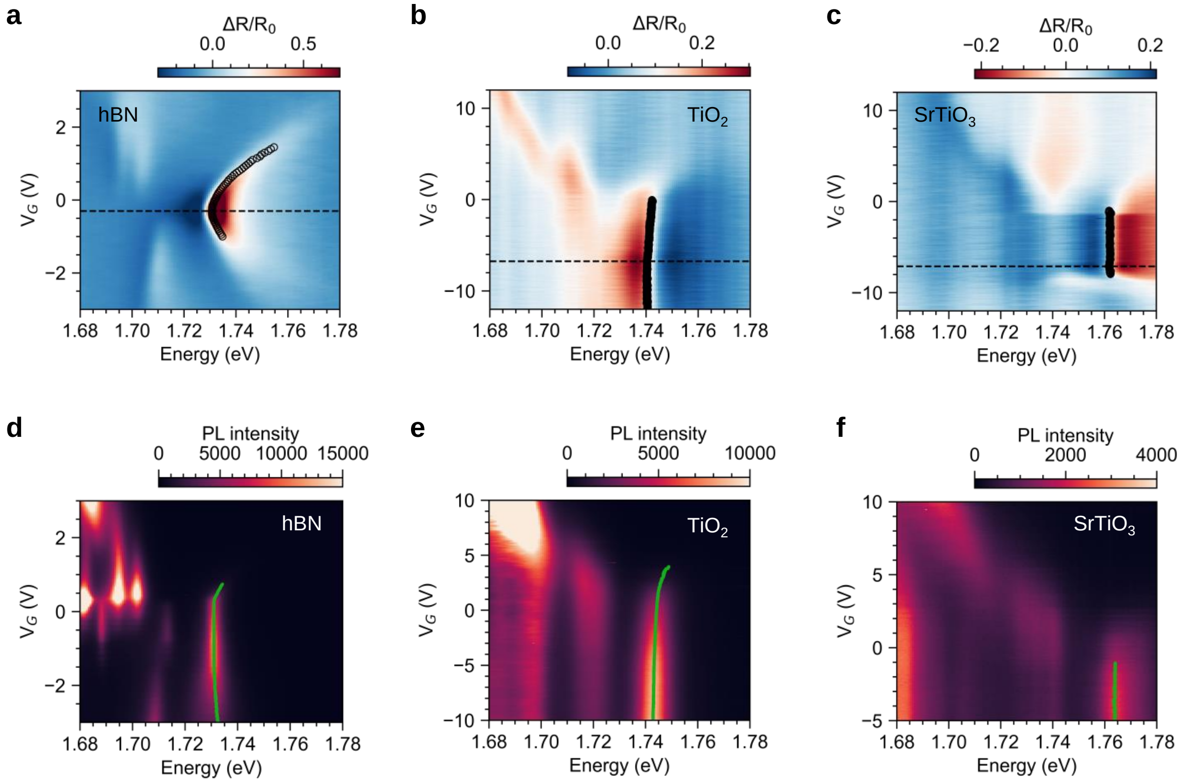

Figure S2. Reflection contrast and PL data analysis. Gate-dependent reflection contrast spectra from the hBN (a), TiO_2 (b), and $SrTiO_3$ (c) device. A dispersive Lorentzian[1] was used to fit X^0 near the charge-neutral region. The energy of the dispersive Lorentzian fit is overlaid (in black). The charge neutrality point is extracted via the minimum of E_X and is indicated by a horizontal dashed line. Gate-dependent PL spectra from the hBN (d), TiO_2 (e), and $SrTiO_3$ (f). A Lorentzian was used to fit X^0. The energy of the Lorentzian fit is overlaid (in green).

S3 Blueshift of X^0 Due to Charge Doping

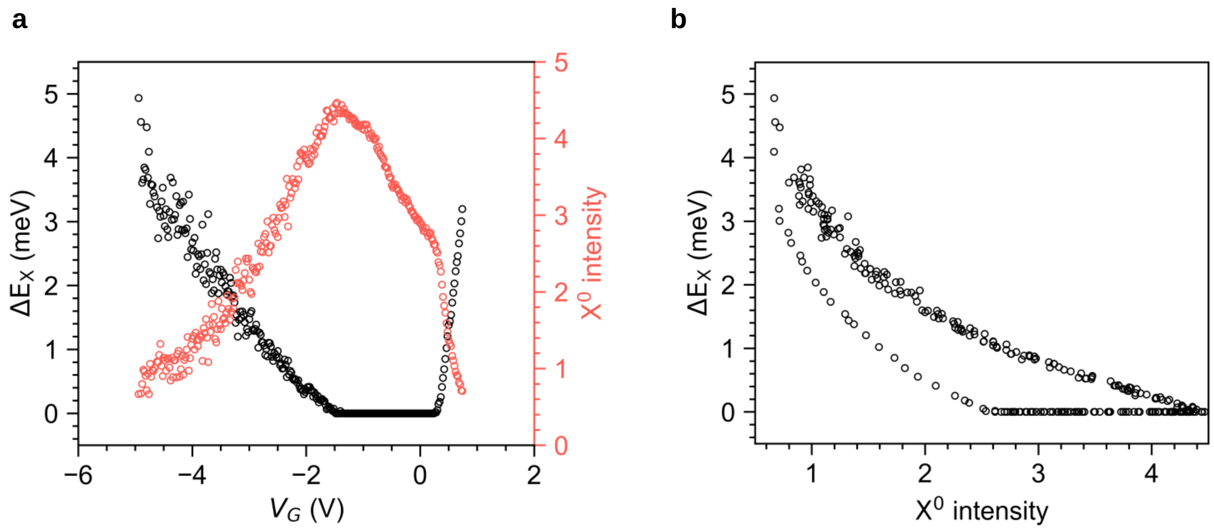

Figure S3. Effect of charge doping on E_X for monolayer WSe_2 in the hBN configuration. (a) E_X change from its value at charge neutrality (black), and X^0 intensity (coral) as a function of the gate voltage (V_G). The data is extracted from the fit discussed in Fig. S2. (b) E_X change from its value at charge neutrality as a function of its intensity. The data shows that at higher charge doping, X^0 loses almost an order of magnitude of its maximum intensity when it has already blueshifted by 5 meV, consistent with Ref.[2]

S4 Rydberg Series of WSe_2 in the hBN Device

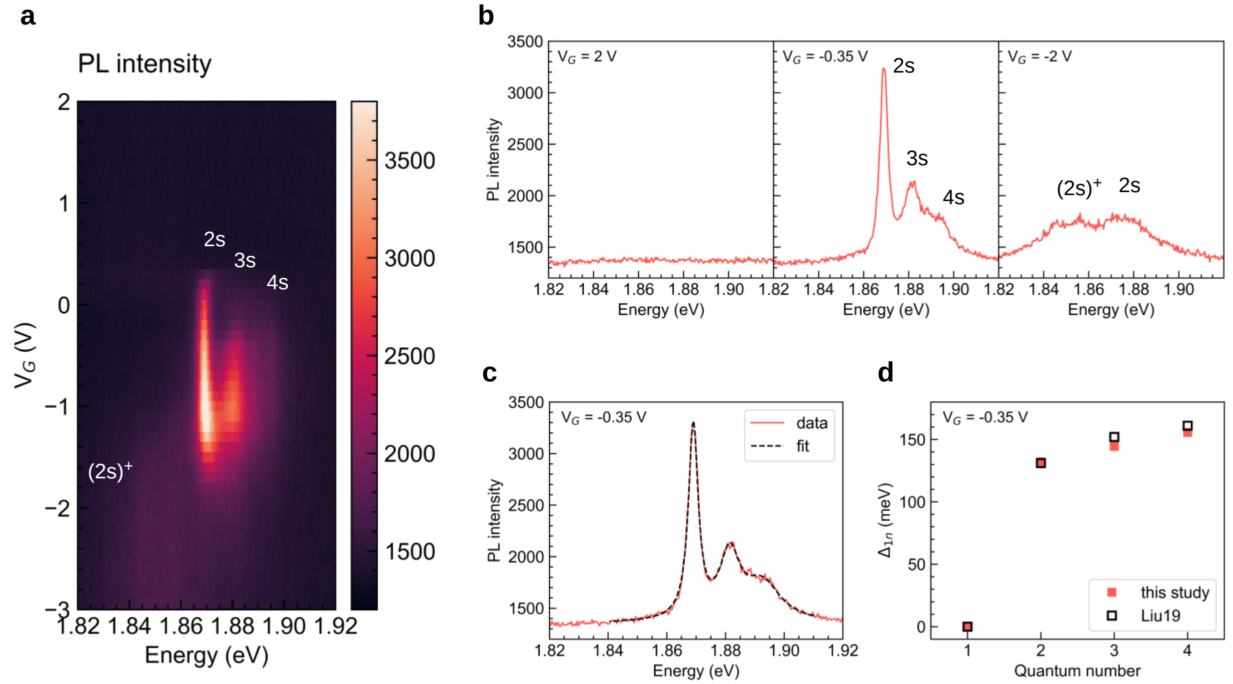

Figure S4. PL of the exciton Rydberg series of monolayer WSe_2 in the hBN configuration. (a) Gate-dependent PL spectra of the hBN device showing a well-resolved exciton Rydberg series of WSe_2, namely the $2s$, $3s$, and $4s$ excitons, in addition to the positively charged $2s$ resonance $(2s)^+$. (b) PL spectra extracted from (a) in three different charge-doping regimes: electron doping (2 V), charge neutrality (-0.35 V), and hole doping (-2 V). (c) Fitting of the Rydberg series using 3 Lorentzians. (d) Energy difference of the nth Rydberg resonance and the $1s$ resonance showing excellent agreement with Ref.,[3] reflecting high sample quality.

S5 $2s$ Exciton in an $SrTiO_3$ Device

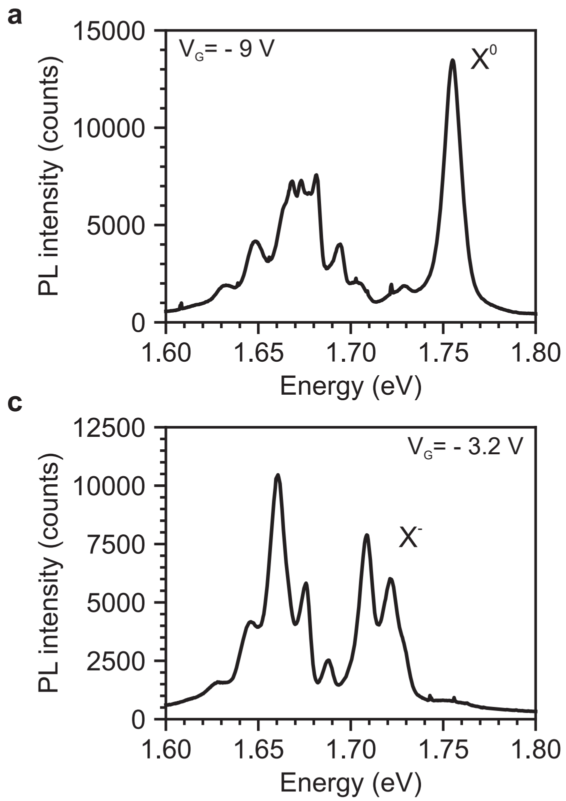

Figure S5. $2s$ exciton in an $SrTiO_3$ device. (a-d) PL spectrum of the $SrTiO_3$ device at different charge configurations. (a) spectrum at charge neutrality, showing a dominant X^0 peak at about 1.755 eV. (b) spectrum at charge neutrality at a higher energy range than (a), showing the $2s$ exciton about ∼1.832 eV. (c) spectrum in the electron doping regime, as witnessed by the presence of the negative trions X^-. (d) spectrum in the electron doping regime and in the same energy range as in (b), showing that the $2s$ exciton peak has disappeared, as expected for a charge-neutral feature.

Figure S5 shows the $1s$ and $2s$ excitons in $SrTiO_3$. While the 1s exciton is blueshifted with respect to the $1s$ exciton in hBN, the $2s$ exciton lies ∼1.832 eV, redshifted about 40 meV compared to the $2s$ exciton in hBN. This is a consequence of the increased delocalization due to the increased radius for excited exciton states. The binding energy of the $2s$ and its change in a different dielectric environment are smaller compared to the $1s$, while the self-energy still contributes to red-shifting the optical resonance. In addition, the weight of the environment outside the TMD increases in the dielectric function, similar to the "slab"

model. As a result, the $2s$ exciton redshifts because the binding energy change is calculated at even larger r than for the $1s$ and, unlike in the $1s$ case, r is now too large for $\varepsilon(0)$ to compensate for the change of the self-energy calculated at $r \rightarrow 0$ even if screened by $\varepsilon(infty)$.

S6 Neutral Exciton Energy Distribution

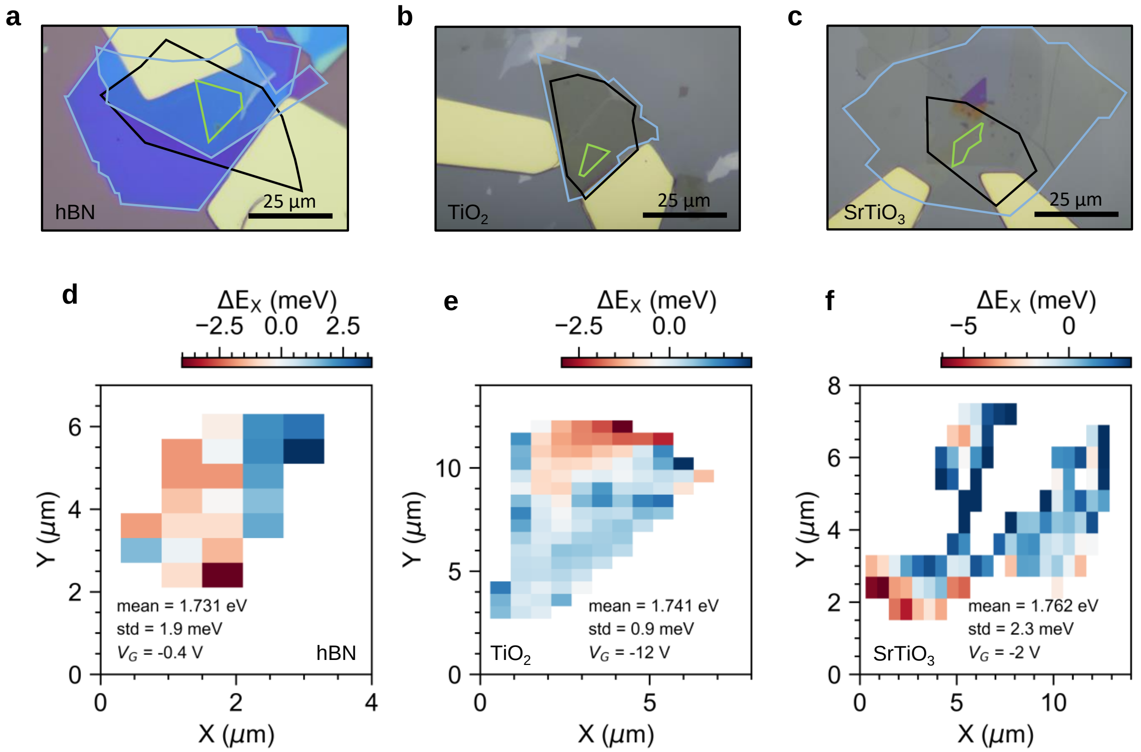

Figure S6. Neutral exciton energy distribution from PL sample maps. (a-c) optical micrographs of the hBN (a), TiO_2 (b), and $SrTiO_3$ (c) devices. (d-f) Two-dimensional PL maps of the E_X variation from the mean energy in the hBN (d), TiO_2 (e), and $SrTiO_3$ (f) devices. A threshold on X^0 PL intensity was set to filter out pixels containing flake edges and electrically unconnected regions of the monolayers. The data shows an E_X standard deviation lower than 3 meV in all devices, an order of magnitude lower than the observed blueshift.

S7 Temperature-dependence of the $SrTiO_3$ Device and Saturation of the Dielectric Effect

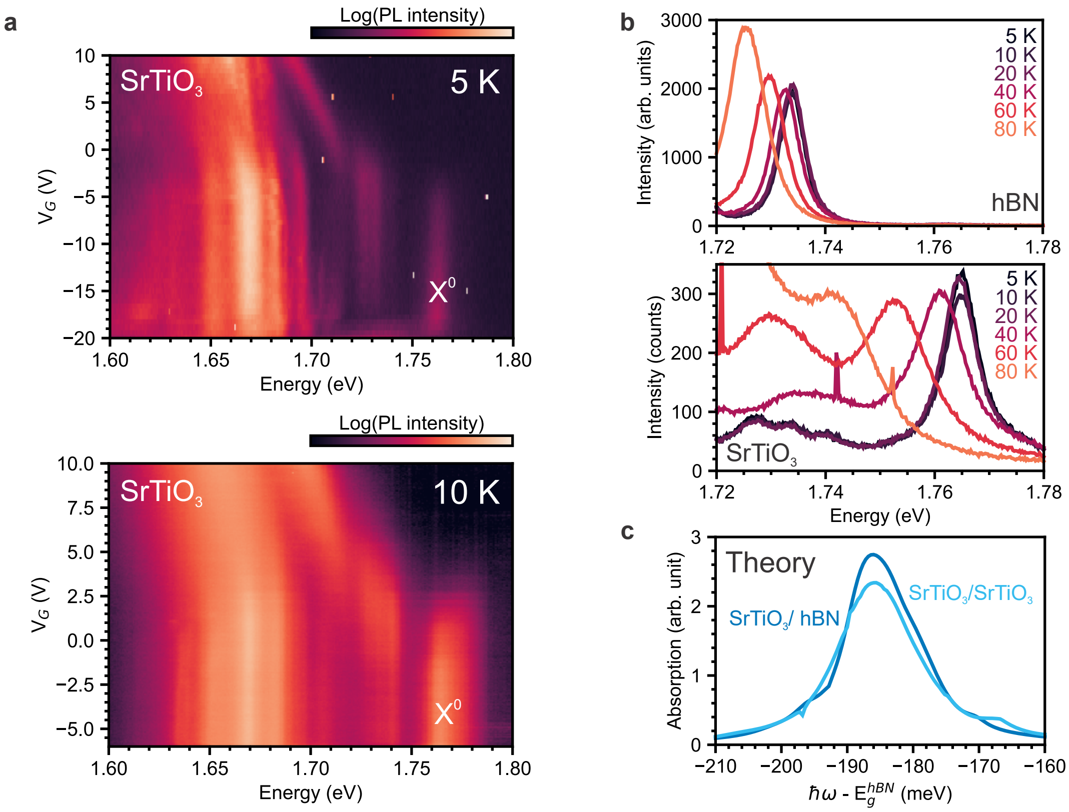

Figure S7. Temperature-dependence of the $SrTiO_3$ device and saturation of the dielectric effect. (a) Gate-dependent PL spectra of monolayer WSe_2 in the $SrTiO_3$ configuration for two different temperatures: 5 K (top panel) and 10 K (bottom panel). The gate-dependent spectra at 10 K are the same as shown in the main text. (b) Temperature-dependent PL spectra of X^0 of the hBN (top panel) and the $SrTiO_3$ device (bottom panel) taken close to charge neutrality at: 5 K, 10 K, 20 K, 40 K, 60 K, and 60 K. (c) Comparison between the calculated absorption spectra of the $SrTiO_3$/hBN device and a hypothetical $SrTiO_3$/$SrTiO_3$ configuration.

Figures S7a and b show temperature-dependent measurements on the hBN and $SrTiO_3$ devices. Although the dielectric constant of $SrTiO_3$ decreases rapidly going from lower temperature to higher temperature,[4] X^0 energy doesn't show a temperature-dependent shift going from 5 K to 10 K (Fig. S7a). Figures S7b compares the temperature-dependent PL spectra of the hBN and $SrTiO_3$ devices. In the hBN configuration, X^0 exhibits almost

constant energy up to 40 K, and it shifts by about 9 meV at 80 K relative to 5 K. This effect stems from the decrease of the band gap at higher temperatures due to thermal effects and is consistent with previous reports.[5] In the $SrTiO_3$ configuration, X^0 exhibits a constant energy up to 20 K, although the dielectric constant of $SrTiO_3$ decreases rapidly as the temperature increases.[4] This suggests a saturation of the dielectric effect: starting from the dielectric constant of $SrTiO_3$ at $\sim$ 20 K), any further increase in the dielectric constant doesn't induce a further X^0 shift. From 20 to 80 K, X^0 exhibits a more significant shift in the $SrTiO_3$ device compared to the hBN device, namely: 4 meV, 12 meV, and more than 20 meV at 40 K, 60 K, and 80 K, respectively. This discrepancy suggests that in addition to the shift stemming from the band gap reduction due to thermal effects, a further mechanism is at play. Starting from 20K, the decrease in the dielectric constant of $SrTiO_3$ when the temperature increases results in an effective decrease in the screening, and thus a redshift of X^0. This further corroborates the blueshifting of X^0 for stronger-screening environments and offers a new route for the tunability of the optoelectronic properties of atomically thin semiconducting materials with applications in nanophotonics.[6] However, a dedicated study would be required to rigorously disentangle the contribution to the X^0 energy shift of the temperature-dependent $\varepsilon(0)$ screening from the thermal shift due to the increased exciton-phonon coupling. Figure S7c shows the calculated absorption spectra for encapsulation in $SrTiO_3$/hBN and $SrTiO_3/SrTiO_3$. The latter spectrum doesn't exhibit any distinguishable shift relative to the former. This may indicate that the latter configuration already constitutes a limiting case of the dynamical screening, or it may instead indicate that the change in the effective r_2 (from ~ 2500 to ~ 4000) is insufficient to cause an appreciable shift.

S8 Theoretical methods

S8.1 Theory

The static Coulomb interaction between charge particles in the monolayer is obtained by solving the Poisson Equation with the appropriate structure geometry. The simulated structure geometry is a monolayer with thickness d sandwiched between top and bottom layers with dielectric constants $\epsilon_{\rm t}$ and $\epsilon_{\rm b}$. The TMD monolayer is modeled as three atomic sheets with polarizabilities χ_+ for the central Tungsten (W) sheet and χ_- for the top and bottom Selenium (Se) ones, displaced by $\pm d/4$ from the center. The model was developed in Ref.[7] and has been employed to study several problems.[8–10] The resulting static potential for the interaction between two charges in the monolayer is

$$V(q) = \frac{2\pi e^2}{A\ \epsilon(q)\ q}, \tag{1}$$

where A is the area of the system, q is the transferred crystal momentum during the interaction, and the static permittivity function of the structure is given by

$$\epsilon(q) = \frac{1}{2}\left[\frac{N_t(q)}{D_t(q)} + \frac{N_b(q)}{D_b(q)}\right]. \tag{2}$$

Defining $p_j \equiv (\epsilon_j - 1)/(\epsilon_j + 1)$ for the top and bottom dielectric constants ($j = b/t$), we get that

$$\begin{aligned} D_j(q) &= 1 + q\ell_- - q\ell_-(1+p_j)\mathrm{e}^{-\frac{qd}{2}} - (1-q\ell_-)p_j\mathrm{e}^{-qd}, \\ N_j(q) &= (1+q\ell_-)(1+q\ell_+) + [(1-p_j) - (1+p_j)\,q\ell_+]\,q\ell_-\mathrm{e}^{-\frac{qd}{2}} + \\ &\quad (1-q\ell_-)(1-q\ell_+)p_j\mathrm{e}^{-qd}. \end{aligned} \tag{3}$$

where $\ell_\pm = 2\pi\chi_\pm$.

The Coulomb potential in Eq. (1) becomes frequency dependent, $V(q) \rightarrow V(q, \omega)$, by using dynamical polarization parameters in Eq. (3). Namely,

$$p_j \rightarrow p_j(\omega) = \frac{\epsilon_j(\omega) - 1}{\epsilon_j(\omega) + 1} , \qquad \text{where} \qquad \epsilon_j(\omega) = \epsilon_\infty \prod_i \frac{\omega^2_{i,j,\mathrm{LO}} - \omega^2}{\omega^2_{i,j,\mathrm{TO}} - \omega^2} . \tag{4}$$

$\epsilon_j(\omega)$ is the frequency-dependent permittivity of the $j = b/t$ layer, as given by Eq. (1) of the main manuscript. The ratio between the static and high-frequency permittivities is the celebrated Lyddane–Sachs–Teller relation $\epsilon_j(\omega = 0)/\epsilon_j(\omega = \infty) \equiv \epsilon_{j,0}/\epsilon_{j,\infty} = \prod_i \omega^2_{i,j,\mathrm{LO}}/\omega^2_{i,j,\mathrm{TO}}$. The index i runs over the optical-phonon modes of the $j = b/t$ layer, where $\omega_{i,j,\mathrm{LO/TO}}$ is the associated frequency of the longitudinal/transverse optical lattice vibration.

The dynamical Coulomb potential has singularities at the phonon frequencies. To circumvent this difficulty when solving the Bethe-Salpeter Equation (BSE) or evaluating the self-energies, we use finite-temperature Green's function formalism in which real frequencies are replaced by imaginary and discrete Matsubara frequencies.[11] Namely, $V(\mathbf{q}, \omega)$ is replaced with $V(\mathbf{q}, z - z')$, where z and z' are imaginary Matsubara energies of fermions before and after the interaction. Their discretized energy form is $(2\ell + 1)\pi i k_\mathrm{B} T$, where ℓ is an integer and T is temperature. Consequently, the positive real number ω^2 in Eq. (4) is replaced by $(z - z')^2$ which is a negative real number. Rather than having singularities, the permittivity function $\epsilon_j(z - z')$ is now monotonously decaying from ϵ_0 to ϵ_∞ as $z - z'$ departs from 0.

S8.2 Dynamical Self-Energy

The self-energy of an electron in the conduction (c) or valence band (v) is calculated from a self-consistent solution of the following Equation

$$\Sigma_i(\mathbf{k}, z) = -\frac{1}{\beta} \sum_{\mathbf{q}, z'} G_i(\mathbf{k} - \mathbf{q}, z') V(\mathbf{q}, z - z'). \tag{5}$$

where $\beta^{-1} = k_B T$, $i = \{\text{c,v}\}$, and the Dyson equation provides the Green's function

$$G_i(\mathbf{k}, z) \;=\; \frac{G_i^0(\mathbf{k}, z)}{1 - G_i^0(\mathbf{k}, z)\Sigma_i(\mathbf{k}, z)} = \frac{1}{z - \varepsilon_i(\mathbf{k}) + \mu - \Sigma_i(\mathbf{k}, z)}. \tag{6}$$

$\mathbf{k}$ and μ are the electron momentum and its chemical potential, respectively. $\varepsilon_{\text{c}}(\mathbf{k}) = E_{\text{g}} + \hbar^2 k^2/2m_c$ is the energy dispersion of the electron in the conduction band and $\varepsilon_{\text{v}}(\mathbf{k}) = \hbar^2 k^2/2m_{\text{v}}$ is the corresponding one in the valence band.

One difficulty of self-energy calculations is the divergence of the sum over $\mathbf{q}$. We illustrate this point by using the non-dynamical bandgap renormalization (BGR), wherein the potential becomes $V(\mathbf{q}, z - z') \to V(\mathbf{q})$ and the sum over Matsubara energies in Eq. (5) is rendered straightforward, $\Sigma_i(\mathbf{k}, z) = \Sigma_i(\mathbf{k}) = \pm\frac{1}{2}\sum_{\mathbf{q}} V(\mathbf{q})$. The $\pm$ denotes the self-energy of an electron (hole) in the conduction (valence) band. The 2D potential $V(q)$ scales as q^{-2} in the short wavelength limit, resulting in a logarithmic divergence of the sum over $\mathbf{q}$. We circumvent this problem by choosing a reference TMD structure with respect to which energy shifts are calculated. Without loss of generality, we choose a reference system whose corresponding potential $V_0(q)$ is evaluated with the static permittivity function, as given by Eqs. (1)-(3), using $\epsilon_{\text{t}} = \epsilon_{\text{b}} = 3.8$. The BGR of a given system with respect to the reference system is then given by

$$\tilde{\Sigma}_i(\mathbf{k}, z) \;=\; -\frac{1}{\beta}\sum_{\mathbf{q}, z'} G_i(\mathbf{k} + \mathbf{q}, z') \times \left[V(\mathbf{q}, z - z') - V_0(\mathbf{q})\right], \tag{7}$$

where the free electron Green's function now becomes

$$G_i(\mathbf{k}, z) = \frac{1}{z - \varepsilon_i(\mathbf{k}) + \mu - \tilde{\Sigma}_i(\mathbf{k}, z)}. \tag{8}$$

The dynamical self-energy $\tilde{\Sigma}_i(\mathbf{k}, z)$ can be self-consistently calculated from Eqs. (7) and (8) using an iterative method.

S8.3 Dynamical Bethe-Salpeter Equation

The BSE is an equation for bound states between two particles. Its dynamical version is used here to describe the interaction between electron and hole excited by light with negligible momentum[12–14]

$$G(\mathbf{k}, z, \Omega) = G^0(\mathbf{k}, z, \Omega) \ + \ \frac{1}{\beta}\sum_{\mathbf{q},z'} G^0(\mathbf{k}, z, \Omega) V(\mathbf{q}, z - z') G(\mathbf{k} + \mathbf{q}, z', \Omega), \tag{9}$$

where the Green's function of a free electron-hole pair is given by

$$G^0(\mathbf{k}, z, \Omega) \ = \ \frac{1}{\Omega - z - \varepsilon_{\mathrm{c}}(\mathbf{k}) - \tilde{\Sigma}_{\mathrm{c}}(\mathbf{k}, \Omega - z) + \mu} \times \frac{1}{z + \varepsilon_{\mathrm{v}}(\mathbf{k}) + \tilde{\Sigma}_{\mathrm{v}}(\mathbf{k}, z) - \mu}. \tag{10}$$

Ω, an even (bosonic) imaginary Matsubara energy, is related to the energy of the photon exciting the electron-hole pair. Equation (9) can be solved using the iterative method, the same method as the one used for calculating the self-energies in Eqs. (7)-(8). One can notice that solutions of different bosonic frequencies Ω are decoupled, and therefore, equations of different Ωs can be solved independently. The solutions are then used to find the contracted pair function

$$g(\mathbf{k}, \Omega) = -\beta^{-1} \sum_{z} G(\mathbf{k}, z, \Omega). \tag{11}$$

The final step is to analytically continue the contracted pair function to the real-frequency axis, $g(\mathbf{k}, \Omega \to \omega + i\delta)$, using the Padé approximation technique.[12,14,15] The real-frequency pair function is related to optical absorption by

$$\mathrm{A}(\omega) = -\sum_{\mathbf{k}} \mathrm{Im}\left[g(\mathbf{k}, \Omega \to \omega + i\delta)\right], \tag{12}$$

where δ is broadening parameter which might include effects of finite exciton lifetime, scattering off impurities, and thermal fluctuations. Note that temperature in this formalism

sets the energy resolution of Matsubara frequencies and is not related to the broadening of resonance peaks which is controlled by δ. In this work, we keep $\delta = 3$ meV for the sake of simplicity.

In the non-dynamical regime (static permittivity), the potential and self-energies are frequency independent. The BSE in Eq. (9) can be further contracted, yielding

$$g(\mathbf{k},\Omega) = g^0(\mathbf{k},\Omega) - \sum_{\mathbf{q}} g^0(\mathbf{k},\Omega)\; V(\mathbf{q})\; g(\mathbf{k}+\mathbf{q},\Omega), \tag{13}$$

and the corresponding function of a free electron-hole pair is given by[11,13]

$$g^0(\mathbf{k},\Omega) \;=\; -\beta^{-1}\sum_{z} G^0(\mathbf{k},z,\Omega) = \frac{f_{\mathrm{v}}(\mathbf{k}) - f_{\mathrm{c}}(\mathbf{k})}{\Omega + \varepsilon_{\mathrm{v}}(\mathbf{k}) + \tilde{\Sigma}_{\mathrm{v}}(\mathbf{k}) - \varepsilon_{\mathrm{c}}(\mathbf{k}) - \tilde{\Sigma}_{\mathrm{c}}(\mathbf{k})}. \tag{14}$$

$f_{\mathrm{c(v)}}(\mathbf{k})$ is the Fermi-Dirac distribution function for electrons in the conduction (valence) band.

S8.4 Distinction between static and dynamical calculations

When dealing with self-energies of free electrons or holes (i.e., when they are not part of an excitonic complex), their calculated self-energies are very close whether they are calculated with frequency-dependent permittivity or with static permittivity using the low-frequency dielectric constant ϵ_0. Namely, free charge particles in the monolayer are also screened by phonons in the polar dielectric materials.

Unlike the case of free charge particles, the self-energy of the electron in the exciton is associated with $(\Omega - z)$ whereas that of the hole with z, as shown by Eq. (10). The bosonic frequency Ω is related to the photon energy, which is of the order of the bandgap energy, a large value compared with phonon or binding energies. Consequently, the self-energy of at least one of the exciton's components asymptotically approaches the value of the self-energy when calculated non-dynamically with ϵ_∞. An alternative view is that the energy difference

between the exciton components is encoded as a time-dependent phase factor $\exp(iE_g t/\hbar)$, which leads to a dominant contribution from the high-frequency part of the dielectric function to the exciton's BGR.

S8.5 Dynamical effects in the optical spectrum

The dynamical potential $V(\mathbf{q}, z-z')$ affects both the BGR and binding energy. To decouple these effects, we focus first on the binding energy by neglecting the self-energy terms in the BSE (i.e., $\tilde{\Sigma}_{\mathrm{c/v}}(\mathbf{k}, z) = 0$ in Eq. (9)). The resulting absorption spectra of hBN-WSe_2-$SrTiO_3$ structures are shown in Fig. 3b of the main text. For comparison, we have also calculated the absorption spectra with static permittivities using low-frequency dielectric constants (red) and high-frequency ones (brown). The exciton binding energy is $E_{\mathrm{b}} = 122$ meV when using the dynamical potential in hBN-WSe_2-$SrTiO_3$. Corresponding values of the non-dynamical calculations are $E_{\mathrm{b}}^0 = 104$ meV and $E_{\mathrm{b}}^{\infty} = 181$ meV. The dynamical binding energy is closer to the one calculated with ϵ_0, meaning that screening of the interaction between the electron and hole is dominated by the low-frequency part of the dielectric function.

Next, we include the self-energy in the calculations of the absorption spectra and consider the competition between BGR and binding energy. The energy blueshift can be recovered if dynamical effects are considered through the potential $V(\mathbf{q}, z-z')$, self-energies $\tilde{\Sigma}_{\mathrm{c}}(\mathbf{k}, \Omega - z)$ and $\tilde{\Sigma}_{\mathrm{v}}(\mathbf{k}, z)$, and if replacing the encapsulating materials involve a large change in ϵ_0 and a small change in ϵ_∞. Figure 3d of the main text shows the resulting absorption spectra of three different structures: hBN-WSe_2-hBN, hBN-WSe_2-TiO_2, and hBN-WSe_2-$SrTiO_3$. In agreement with the experimental results, replacing the supporting hBN layer with TiO_2 leads to energy blueshift, which is further increased when $SrTiO_3$ is used as support. The opposite energy-shift trends of calculations with static and dynamical permittivities can be explained as follows. The binding energy is mainly dominated by the low-frequency part of the dielectric function, where the change is from $\epsilon_0^{\mathrm{hBN}} = 4.9$ to $\epsilon_0^{\mathrm{TiO_2}} \sim 200$ and then to

$\epsilon_0^{\mathrm{SrTiO_3}} = 25000$. As a result, the change in binding energy is relatively significant. On the other hand, the self-energies of the electron and hole in the exciton have larger contribution from the high-frequency part, where the change is from $\epsilon_\infty^{\mathrm{hBN}} = 3.8$ to $\epsilon_\infty^{\mathrm{TiO_2}} \sim \epsilon_\infty^{\mathrm{SrTiO_3}} \sim 6$. As a result, the BGR effect is relatively mitigated. The confluence of both trends is that the energy redshift from BGR is smaller than the energy blueshift from binding energy ($\Delta E_{\mathrm{g}} < |\Delta E_{\mathrm{b}}|$), leading to overall energy blueshift of the exciton resonance.

S8.6 Iterative method

Equations (7)-(8) for electron/hole self-energy and Eq. (9) for the exciton Green's function are solved by the iterative method. The procedure starts with a guessed solution, which is the static self-energy for Eqs. (7)-(8) and the Green's function of the free electron-hole pair for Eq. (9). The guessed solution is then inserted into the right-hand side of the equations to calculate the new values of the quantities on the left-hand side. These new values are again inserted into the right-hand side and the process is repeated until convergence is reached (for more information, please see the Appendix B of Ref.[16])

S8.7 Material parameters

Table 1. **Dielectric parameters used in the calculations.**

Materials	hBN	HfO_2	TiO_2	$SrTiO_3$
ϵ_0	4.9[7,17,18]	15[19,20]	192[21,22]	25000[4,23–26]
ϵ_∞	3.8[7,17,18]	5[19,20]	6[21,22]	6[4,23–26]
$r_2 = \epsilon_0/\epsilon_\infty$	1.3	3	3	4000

The dielectric parameters used in the calculations are summarized in Table 1. The following parameters are used for the WSe_2 monolayer in different dielectric environments: (i) The effective masses are $m_c = 0.29\ m_0$ (top conduction band valley), $m_v = 0.36\ m_0$ (top valence-band valley).[27] The kinetic energies of electrons and holes are evaluated by parabolic

energy dispersion.

(ii) The monolayer parameters of the potential are $d = 6$ A and $l_+ = l_- = 5d$ (Appendix A of Ref.[16]).

(iii) The dielectric constants of hBN, TiO_2, and $SrTiO_3$, and the parameter $r_1 = \hbar\omega_{TO}/\epsilon_{X^0}$ (with $\epsilon_{X^0} = 170$ meV), are listed in Table 1. (iv) The following parameters are used in the numerical calculations of the self-energy and BSE: $n_K = 100$, $E^k_{Cut} = 2$ eV,$n_Z = 3200$, $T = 80$ K, and the broadening used in the analytical continuation of Eqs. (21) and (24) in Ref.[16] is $\delta = 3$ meV.

References

(1) Smoleński, T.; Dolgirev, P. E.; Kuhlenkamp, C.; Popert, A.; Shimazaki, Y.; Back, P.; Lu, X.; Kroner, M.; Watanabe, K.; Taniguchi, T.; Esterlis, I.; Demler, E.; Imamoğlu, A. Signatures of Wigner crystal of electrons in a monolayer semiconductor. *Nature* **2021**, *595*, 53–57.

(2) Van Tuan, D.; Scharf, B.; Wang, Z.; Shan, J.; Mak, K. F.; Žutić, I.; Dery, H. Probing many-body interactions in monolayer transition-metal dichalcogenides. *Phys. Rev. B* **2019**, *99*, 085301.

(3) Liu, E.; van Baren, J.; Taniguchi, T.; Watanabe, K.; Chang, Y.-C.; Lui, C. H. Magnetophotoluminescence of exciton Rydberg states in monolayer WSe_2. *Phys. Rev. B* **2019**, *99*, 205420.

(4) Neville, R. C.; Hoeneisen, B.; Mead, C. A. Permittivity of Strontium Titanate. *Journal of Applied Physics* **1972**, *43*, 2124–2131.

(5) Nagler, P.; Ballottin, M. V.; Mitioglu, A. A.; Durnev, M. V.; Taniguchi, T.; Watanabe, K.; Chernikov, A.; Schüller, C.; Glazov, M. M.; Christianen, P. C.; Korn, T. Zeeman Splitting and Inverted Polarization of Biexciton Emission in Monolayer WS_2. *Phys. Rev. Lett.* **2018**, *121*, 057402.

(6) Weber, T.; Kühner, L.; Sortino, L.; Ben Mhenni, A.; Wilson, N. P.; Kühne, J.; Finley, J. J.; Maier, S. A.; Tittl, A. Intrinsic strong light-matter coupling with self-hybridized bound states in the continuum in van der Waals metasurfaces. *Nat. Mater.* **2023**, *22*, 970–976.

(7) Van Tuan, D.; Yang, M.; Dery, H. Coulomb interaction in monolayer transition-metal dichalcogenides. *Phys. Rev. B* **2018**, *98*, 125308.

(8) Van Tuan, D.; Shi, S.-F.; Xu, X.; Crooker, S. A.; Dery, H. Six-Body and Eight-Body Exciton States in Monolayer WSe_2. *Phys. Rev. Lett.* **2022**, *129*, 076801.

(9) Van Tuan, D.; Dery, H. Composite excitonic states in doped semiconductors. *Phys. Rev. B* **2022**, *106*, L081301.

(10) Van Tuan, D.; Jones, A. M.; Yang, M.; Xu, X.; Dery, H. Virtual Trions in the Photoluminescence of Monolayer Transition-Metal Dichalcogenides. *Phys. Rev. Lett.* **2019**, *122*, 217401.

(11) Mahan, G. D. *Many-particle physics*, third edition ed.; Physics of solids and liquids; Springer Science + Business Media, LLC: New York, 2000.

(12) Scharf, B.; Tuan, D. V.; Žutić, I.; Dery, H. Dynamical screening in monolayer transition-metal dichalcogenides and its manifestations in the exciton spectrum. *J. Phys.: Condens. Matter* **2019**, *31*, 203001.

(13) Haug, H.; Schmitt-Rink, S. Electron theory of the optical properties of laser-excited semiconductors. *Progress in Quantum Electronics* **1984**, *9*, 3–100.

(14) Van Tuan, D.; Scharf, B.; Žutić, I.; Dery, H. Marrying Excitons and Plasmons in Monolayer Transition-Metal Dichalcogenides. *Phys. Rev. X* **2017**, *7*, 041040.

(15) Vidberg, H. J.; Serene, J. W. Solving the Eliashberg equations by means ofN-point Padé approximants. *J Low Temp Phys* **1977**, *29*, 179–192.

(16) Tuan, D. V.; Dery, H. Effects of dynamical dielectric screening on the excitonic spectrum of monolayer semiconductors. 2024.

(17) Cai, Y.; Zhang, L.; Zeng, Q.; Cheng, L.; Xu, Y. Infrared reflectance spectrum of BN calculated from first principles. *Solid State Communications* **2007**, *141*, 262–266.

(18) Dai, S. et al. Tunable Phonon Polaritons in Atomically Thin van der Waals Crystals of Boron Nitride. *Science* **2014**, *343*, 1125–1129.

(19) Bright, T. J.; Watjen, J. I.; Zhang, Z. M.; Muratore, C.; Voevodin, A. A. Optical properties of HfO2 thin films deposited by magnetron sputtering: From the visible to the far-infrared. *Thin Solid Films* **2012**, *520*, 6793–6802.

(20) Hsain, H. A.; Lee, Y.; Materano, M.; Mittmann, T.; Payne, A.; Mikolajick, T.; Schroeder, U.; Parsons, G. N.; Jones, J. L. Many routes to ferroelectric HfO2: A review of current deposition methods. *Journal of Vacuum Science Technology A* **2022**, *40*, 010803.

(21) Parker, R. A. Static Dielectric Constant of Rutile (TiO_2), 1.6-1060˚K. *Phys. Rev.* **1961**, *124*, 1719–1722.

(22) Schöche, S.; Hofmann, T.; Korlacki, R.; Tiwald, T. E.; Schubert, M. Infrared dielectric anisotropy and phonon modes of rutile TiO2. *Journal of Applied Physics* **2013**, *113*, 164102.

(23) Sawaguchi, E.; Kikuchi, A.; Kodera, Y. Dielectric Constant of Strontium Titanate at Low Temperatures. *J. Phys. Soc. Jpn.* **1962**, *17*, 1666–1667.

(24) Akimov, I. A.; Sirenko, A. A.; Clark, A. M.; Hao, J.-H.; Xi, X. X. Electric-Field-Induced Soft-Mode Hardening in $SrTiO_3$ Films. *Phys. Rev. Lett.* **2000**, *84*, 4625–4628.

(25) Evarestov, R. A.; Blokhin, E.; Gryaznov, D.; Kotomin, E. A.; Maier, J. Phonon calculations in cubic and tetragonal phases of $SrTiO_3$: A comparative LCAO and plane-wave study. *Phys. Rev. B* **2011**, *83*, 134108.

(26) Sirenko, A. A.; Bernhard, C.; Golnik, A.; Akimov, I. A.; Clark, A. M.; Hao, J. H.; Xi, X. X. Soft-Mode Phonons in SrTiO3 Thin Films Studied by Far-Infrared Ellipsometry and Raman Scattering. *MRS Online Proceedings Library* **1999**, *603*, 245–250.

(27) Kormányos, A.; Burkard, G.; Gmitra, M.; Fabian, J.; Zólyomi, V.; Drummond, N. D.;

Fal'ko, V. k·p theory for two-dimensional transition metal dichalcogenide semiconductors. *2D Mater.* **2015**, *2*, 022001.
